# Changing Seasonality of Influenza in the Post-COVID Era in Japan

**DOI:** 10.31662/jmaj.2023-0150

**Published:** 2023-11-16

**Authors:** Keita Wagatsuma, Teruhime Otoguro

**Affiliations:** 1 Division of International Health (Public Health), Graduate School of Medical and Dental Sciences, Niigata University, Niigata, Japan; 2 Japan Society for the Promotion of Science, Tokyo, Japan; 3 Infectious Diseases Research Center of Niigata University in Myanmar (IDRC), Niigata, Japan

**Keywords:** Seasonality, Influenza, COVID-19, Epidemics, Japan

To the Editor: The onset of the coronavirus disease (COVID-19) pandemic has transformed our policies and behaviors, even our economy and education, and continues to have a threatening impact on people’s health even today ^(1)^. In this context, it is necessary to emphasize that the dynamics of seasonal influenza, which is more common and burdensome in Japan, have also been considerably affected.

With Japan’s abandonment of pandemic control measures, the resurgence of seasonal influenza as a major public health concern now appears inevitable. As a practical matter, notifications of influenza across Japan have surged considerably earlier than usual, reaching a record high at the time of writing, after three years of relative silence (Figure 1). This surge has prompted school closures in multiple prefectures, notably with 22,111 cases reported during the week of September 15, 2023, a staggering 166-fold increase compared to the 133 cases reported in the same period a year earlier ^(2)^. This surge has led to the closure of 10 school buildings and the suspension of classes in 627 schools nationwide. Notably, more than 90.0% of these cases were attributed to influenza A/H3N2, a strain known to cause severe epidemics.

**Figure 1.**
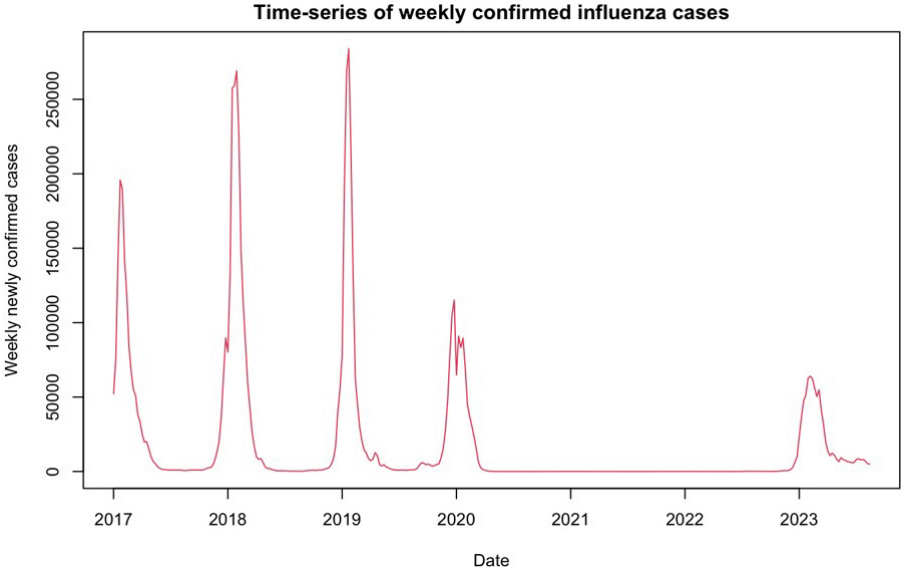
Time series of weekly confirmed influenza cases in Japan from 2017 to the present. The notifications of influenza used in this study were obtained from the Infectious Disease Weekly Report, which was sourced from the National Epidemiological Surveillance of Infectious Diseases data published by the National Institute of Infectious Diseases, Japan under the Ministry of Health, Labor and Welfare, Japan (https://www.niid.go.jp/niid/en/survaillance-data-table-english.html).

The resurgence of seasonal influenza epidemics following a pandemic has also been observed in other regions, such as the United States and Australia, highlighting that Japan’s experience is not unique ^(3)^. The sharp increase in cases can be attributed to the relaxation of measures due to the pandemic and the decrease in the opportunity to acquire antibodies via natural infection; consequently, the level of population immunity against the currently prevalent virus is likely significantly lower than before ^(4)^. This situation may be exacerbated by children under five years of age who have never been exposed to influenza and by individuals at a high risk of severe illness, including older adults and pregnant women. The occurrence of new outbreaks may further strain healthcare resources and lead to delays in diagnosis.

Herein, we report a substantial outbreak of seasonal influenza in Japan that began in the summer of 2023. In Japan, this season’s vaccine supply is adequate, with approximately 16.6 million doses expected to be available as of September 2023; therefore, early vaccination is recommended due to potential early and larger outbreaks ^(5)^. These observations offer valuable insights into the repercussions of COVID-19-related disruptions and highlight the importance of concerted efforts to unravel the knowledge gaps that remain despite years of research.

## Article Information

### Conflicts of Interest

None

### Author Contributions

Both authors conceptualized the manuscript. KW wrote the original draft and TO critically edited it. Both authors read and approved the final version of the manuscript.

### Approval by Institutional Review Board (IRB)

Not applicable.
